# Effect of Nano-CuO on Engineering and Microstructure Properties of Fibre-Reinforced Mortars Incorporating Metakaolin: Experimental and Numerical Studies

**DOI:** 10.3390/ma10101215

**Published:** 2017-10-23

**Authors:** Amir Ghanei, Faezeh Jafari, Mojdeh Mehrinejad Khotbehsara, Ehsan Mohseni, Waiching Tang, Hongzhi Cui

**Affiliations:** 1 Department of Civil Engineering, Mahmoudabad Branch, Islamic Azad University, Mahmoudabad 4645655111, Iran; Ghaneiamir91@gmail.com; 2 Department of Civil Engineering, Malayer University, Malayer 65719-95863, Iran; faeze_jafari666@yahoo.com; 3 Centre for Future Materials (CFM), School of Civil Engineering and Surveying, University of Southern Queensland, Toowoomba QLD 4350, Australia; mojdeh.Mehrinejad@usq.edu.au; 4 School of Architecture and Built Environment, The University of Newcastle, Callaghan NSW 2308, Australia; Ehsan.Mohseni@uon.edu.au; 5 Shenzhen Durability Center for Civil Engineering, Shenzhen University, Shenzhen 518060, China; h.z.cui@szu.edu.cn

**Keywords:** nano-CuO, metakaolin, fibre-reinforced cement mortar, microstructure properties, interfacial transition zone (ITZ), ANFIS method, Pegasos algorithm

## Abstract

In this study, the effects of nano-CuO (NC) on engineering properties of fibre-reinforced mortars incorporating metakaolin (MK) were investigated. The effects of polypropylene fibre (PP) were also examined. A total of twenty-six mixtures were prepared. The experimental results were compared with numerical results obtained by adaptive neuro-fuzzy inference system (ANFIS) and Primal Estimated sub-GrAdient Solver for SVM (Pegasos) algorithm. Scanning Electron Microscope (SEM) was also employed to investigate the microstructure of the cement matrix. The mechanical test results showed that both compressive and flexural strengths of cement mortars decreased with the increase of MK content, however the strength values increased significantly with increasing NC content in the mixture. The water absorption of samples decreased remarkably with increasing NC particles in the mixture. When PP fibres were added, the strengths of cement mortars were further enhanced accompanied with lower water absorption values. The addition of 2 wt % and 3 wt % nanoparticles in cement mortar led to a positive contribution to strength and resistance to water absorption. Mixture of PP-MK10NC3 indicated the best results for both compressive and flexural strengths at 28 and 90 days. SEM images illustrated that the morphology of cement matrix became more porous with increasing MK content, but the porosity reduced with the inclusion of NC. In addition, it is evident from the SEM images that more cement hydration products adhered onto the surface of fibres, which would improve the fibre–matrix interface. The numerical results obtained by ANFIS and Pegasos were close to the experimental results. The value of R^2^ obtained for each data set (validate, test and train) was higher than 0.90 and the values of mean absolute percentage error (MAPE) and the relative root mean squared error (PRMSE) were near zero. The ANFIS and Pegasos models can be used to predict the mechanical properties and water absorptions of fibre-reinforced mortars with MK and NC.

## 1. Introduction

The usage of supplementary cementitious materials (SCMs) has been prevalent in concrete in an effort to decrease the amount of cement while improving the properties of concrete. Application of metakaolin (MK) has been recently developed as SCMs, but its production differs from those produced as residues of industrial process, such as fly ash, microsilica, and blast-furnace slag. In fact, MK is generated under attentively controlled conditions [1]. The calcination processes of kaolinit clay lead to the production of MK, Al2Si2O7 or AS2. Previous research on MK shows that it is an excellent pozzolanic material due to its positive effect on long-term durability of concrete [2]. With regards to workability and setting time, specimens incorporating MK usually required more superplasticizer, since the inclusion of MK steps down the setting time of pastes when compared to the control sample. It has been argued that using MK as a cement replacement may increase the total cost of concrete. However, it is noteworthy that this drawback can be outweighed by the benefits of enhanced durability and strength [1].

Nowadays, different kinds of fibres like steel, glass, synthetic and some natural fibres are commonly used for various applications [3]. Nili et al. [4] investigated the impact resistance and mechanical properties of hooked steel fibre-reinforced concrete. It was reported that the incorporation of steel fibres improved the strength performance of concrete, particularly the splitting tensile and the flexural strengths. In addition, an increase in ductility of the resulting concrete was remarkable. In particular, polypropylene (PP) fibres have attracted a great attention for use as reinforcement in concrete. This is mainly due to their high biological and chemical resistance, particularly in concrete’s high alkaline environment. In addition, PP fibres are relatively low cost and possess various excellent properties, such as low modulus of elasticity, high strength, eminent durability and excellent ductility and anti-cracking performance.

The performance of reinforced concrete is based on the interaction between fibres and cement matrix in which the geometry and properties of fibre play a significant role. However, the contribution of PP in concrete strength improvement is contradictory, as reported in the literature. Richardson concluded that the compressive strength of concrete significantly reduced with the addition of PP fibres [5]. However, Mohseni et al. [6] reported the compressive strength of mortar samples increased with PP fibres content. Alhozaimy et al. [7] stated that PP fibres did not have any considerable effect on compressive strength of concrete. Nevertheless, the research on the interaction of PP fibres and MK and the interfacial transition zone (ITZ) of samples containing both PP fibres and MK on properties of cement mortars, especially the flexural strength is very limited and the results might be different from studying PP and other pozzolans such silica fume or fly ash.

Recently, nanomaterial technology has attracted great attention in virtue of its significant performance in improving the properties of cementitious materials. Many research studies have been carried out, and consistently indicate that the inclusion of nanomaterials could lead to significant improvements in various properties of mortar and concrete. They showed that nanoparticles can alleviate the strength loss caused by other supplementary cementitious materials. For instance, Mohseni et al. [8] studied the binary and ternary blended mortar containing nanoparticles and fly ash and their results indicated that the inclusion of nanoparticles and FA resulted in high early age strength and long-term strength of mortar. However, most of the previous studies were mainly focused on nano-SiO_2_ [9,10,11], nano-TiO_2_ [12,13] and other metal-containing nanoparticles [6,14,15,16,17]. There are very limited studies on nano-copper oxides (nano-CuO) and their effects on physical and mechanical properties of mortar and concrete [18,19,20]. Nano-CuO (NC) has been reported as an active nanoparticle with good potential to improving the strength and thermal properties of cementitious materials. Nazari et al. [18] investigated the effect of CuO nanoparticles on compressive strength of self-compacting concrete. Their results indicated that the compressive strength of samples increased with the NC content up to 4%. Khotbehsara et al. [19] conducted an experimental study on durability of self-compacting mortar containing NC and FA. They showed that the best results for electrical resistivity and chloride permeability were observed in the mixture containing 4% NC and 25% fly ash.

Neural network is currently used in conjunction with laboratory tests to predict concrete strength [21,22]. Cüneyt et al. [23] used adaptive neuro-fuzzy inference system (ANFIS) to estimate the elastic modulus of normal and high strength concrete. The results showed that the ANFIS method outperformed other models in terms of prediction capability. Na et al. [24] applied ANFIS to predict compressive strength of concrete in the range of 6.3–107.7 MPa from 3 to 365 days. Neshat et al. [25] compared the results of ANFIS model with Fuzzy Expert System (FES) to evaluate concrete mixture design. In their research, the slump, maximum size of aggregate (D_max_), concrete compressive strength and fineness modulus were input as layers, and the quantities of water, cement, fine and course aggregate were considered as output layers. Comparison of the results between FES and ANFIS systems indicated that ANFIS model was performed better for both training and prediction than FES [25]. Nazari and Riahi [26] used ANFIS to predict the percentage of water absorption of geopolymers made from seeded fly ash and rice husk bark ash. Fly ash, rice husk ash, temperature of curing and age were selected as input layers, and the predicted value of percentage of water absorption for each cementitious sample was considered as output layer. The results showed that ANFIS has the ability to predict the strength values completely similar to the experimental values, and the R^2^ for training and testing sets were 0.9941 and 0.9929, respectively. Ahmadi [27] used ANFIS and optimal nonlinear regression models to predict elastic modulus of normal and high strength concretes. ANFIS was reported a reliable method to predict concrete strength as there was a good alignment between the experimental data and elastic modulus estimated from the model. Yuan et al. [28] studied the impact of unstructured and structured factors on concrete quality using two hybrid models of genetic based algorithm and ANFIS. The results indicated that both models showed excellent performance in strength prediction. Vakhshouri and Nejadi used ANFIS to predict compressive strength of self-compacting lightweight concrete [29]. In their research, an artificial intelligence was applied as a basic approach to simulate the non-linear and complex behaviour of concrete. The results indicated good predictions using the ANFIS analysis.

The support vector machine (SVM) is a relatively new Artificial Intelligence technique which has been widely applied to solve engineering problems and has yielded encouraging results. Cevik et al. [30] showed that the SVM approach has a comparable or higher performance than traditional learning machines and can be utilized as a powerful tool to solve classification and regression problems. Recently, SVM has been used in predicting concrete properties [31,32]. For instance, Naseri et al. [31] used Wavelet Weighted Least Square SVM (WWLSSVM) and Least Square SVM (LSSVM) models to predict experimental results. Besides, Shalvet et al. [33] introduced Primal Estimated sub-GrAdient Solver for SVM (Pegasos) as a machine learning algorithm to improve the accuracy of estimate by assigning weights for training samples. It is believed that Pegasos is a practical and useful method, however, it has never been tested and compared with other prediction methods in predicting various concrete properties.

To further extend the application of NC in cement-based structures, the effects of NC on early and later age properties of fibre reinforced mortars containing MK were examined in this study. The ordinary Portland cement (OPC) herein was partially replaced by MK at various replacement ratios, along with different dosages of NC and PP fibres used. In this study, the compressive strength, flexural strength and water absorption properties were determined and the influences of NC and MK on these properties are discussed. Therefore, the aim of this research was to investigate the effects of ternary combination of PP fibres, NC and MK on mortar properties, and then compared the experimental results to those predicted by ANFIS and Pegasos models.

## 2. Methodology

### 2.1. Experimental Program

#### 2.1.1. Materials

**Figure 1:**
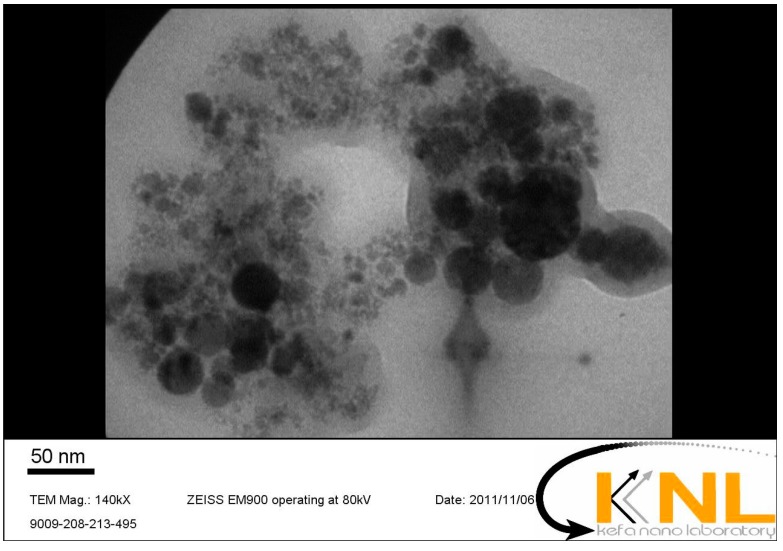
Nano-CuO particles of uniform distribution observed using transmission electron micrographs (TEM) (of size 50 nm).

Natural river sand and ordinary Portland cement type I were used to cast the mortar specimens. MK with average particles size of 8 µm was used as a pozzolanic material with cement. The chemical composition and physical characteristics of cement and MK are given in Table 1. NC in dispersed suspension form with an average particle size of 20 nm was used in this study. The physical properties of nanoparticles are given in Table 2. Figure 1 shows the distribution of NC particles in a micrograph taken by transmission electron micrographs (TEM, EM900, ZEISS, Tehran, Iran). It can be seen from the figure that the NC particles are of spherical shape. To achieve the desired fluidity and appropriate dispersion of nanoparticles, a polycarboxylate type superplasticizer (SP) according to ASTM C494 with a density of 1.03 g/cm^3^ was utilized. The content of superplasticizer was adjusted to keep the same fluidity of samples.

In this study, PP fibres produced from recycled raw materials was chosen due to their superior performances of high resistance to chemical leaching and corrosion, high resilience against impact and freezing, and significant environmental benefits. The properties of PP fibres are shown in Table 3. The fibres had a length of 6 mm and a diameter of 20 micron making an aspect ratio of 300.

#### 2.1.2. Mix Proportions

Twenty-six mixtures were prepared with different amounts of MK, NC, PP fibres and SP. The percentages of MK used were varied between 0% and 30% by weight of the total binder. NC were added to the mixture in amounts of 0%, 1%, 2% and 3% by weight of binder. The PP fibres were used at the rates of 0% and 0.3% of the binder. The amounts of SP added were between 0.2% and 0.94% by weight of the binder. The water to binder ratio (w/b) was kept constant at 0.49 for all the mixtures. Table 4 shows the mix proportions of the mortars in details. In labelling of the mixtures, the number after MK, NC and PP represents the percentages of metakaolin, nano-CuO and polypropylene fibres added into the mortar, respectively. The control mixture (CO) without any additives was also studied.

#### 2.1.3. Production of Specimens

Due to its extremely high fineness, nanoparticles have tendency to show agglomeration in dry state and may not get uniformly distributed in the mix [14]. The mixing and casting of the mortar samples were carried out in accordance with ASTM C305. It is well known that the dispersion of fine particles directly affects the mechanical and physical properties of mortar, so the following mixing procedures were followed to facilitate a uniform distribution of the nano materials: First, the cement and MK were dry-mixed in the mixer at a moderate speed (80 rpm) for 1 min. Then, the mixture were mixed at a high speed (120 rpm) for 90 s with the addition of CuO nanoparticles, fibre and 90% of water. Afterwards, the sand was gradually added over a period of 30 s, while the mixer was running at a medium speed. Then, both remaining water and the superplasticizer were added and stirred at a high speed for 30 s. Finally, the mixture was allowed to rest for 90 s and followed by additional mixing at a high speed for 1 min. The above-mentioned mixing procedures were followed to assist the distribution of nanoparticles and fibres in the mortar.

Fresh mixtures were cast into 50 × 50 × 50 mm^3^ cubes for water absorption, compressive and SEM tests, and 50 × 50 × 200 mm^3^ steel moulds for flexural test. The mortar samples were compacted using a temping rod to exclude the air bubbles from the mortar. The mixtures were demoulded 24 h after casting and then they were cured in water at 23 ± 3 °C until they were tested.

#### 2.1.4. Test Procedures

Compressive strength test was accomplished conforming to ASTM-C109 using a hydraulic testing machine at a loading rate of 1350 N/s. Furthermore, centre-point loading was used for the flexural test with a span of 180 mm and a loading rate of 44 N/s. The compressive and flexural strength tests were conducted on samples at 28 and 90 days. Each test result was the average of three repetitive test specimens.

The water absorption test was performed on mortar sample at 28 days according to the ASTM C642. Saturated surface dry sample was retained in an oven at a temperature of 110 °C for 72 h. The specimens were immersed in water for 72 h after measuring the original weight. Then, the final weight was measured and the absorption was calculated to evaluate the permeability behaviour of the mortar specimens. The absorption test result was the average of two repetitive test specimens.

Scanning electron microscope (SEM, AIS2100, SERONTECHNOLOGIES, Tehran, Iran) was used to analyse the morphology of cement mortar at 28 days. SEM sample was prepared and then dried in the oven at 100 °C for 24 h. The SEM analysis was carried out once the oven-dried sample was cooled down to the room temperature. The samples were then sprayed with gold prior to observation.

### 2.2. Prediction Method

#### 2.2.1. Application of ANFIS to Predict Concrete Properties

An adaptive neuro-fuzzy inference system is categorized as artificial neural network, which is based on the Takagi–Sugeno fuzzy inference system, and its initial application was in the early 1990s [34]. This technique exploited both neural networks and fuzzy logic principles for approximate nonlinear functions [35]. To describe the architecture of an ANFIS, the first order Sugeno-style fuzzy inference system (FIS) is introduced. A first-order Sugeno-style FIS model is a system that manages the process of mapping from a given crisp input to a crisp output, using a fuzzy set theory. The IF-THEN rules were obtained as Equations (1) and (2). Suppose that the base rule of ANFIS contains two fuzzy IF–THEN rules of Takagi and Sugeno’s type.

(1) $$\mathrm{If}\;x_{1}\;\mathrm{is}\;A_{1},x_{2}\;\mathrm{is}\;A_{2},\ldots \;\ldots x_{n}\;\mathrm{is}\;A_{n}$$

(2) $$\mathrm{then}\;y=k_{0}+k_{1}x_{1}+k_{2}x_{2}+\cdots +k_{n}x_{n}$$

where $x_{1},x_{1},\ldots ,x_{n}$ are considered as input variables; $A_{1},A_{2},\ldots ,A_{n}$ are fuzzy sets; and y is the output variable. It is found that in such type of fuzzy rule the output variable is a first-order polynomial on input variables.

**Figure 2:**
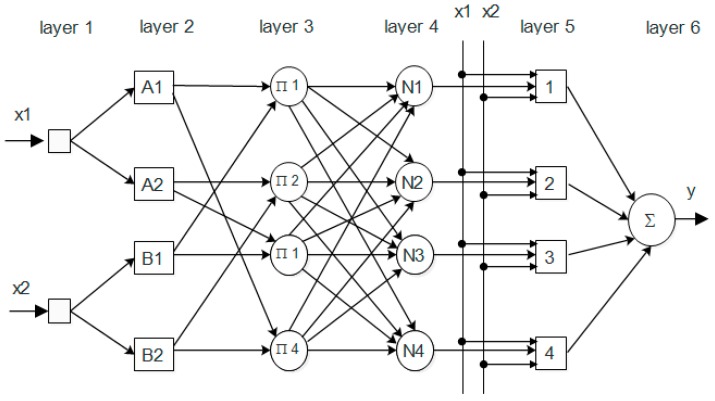
Adaptive neuro-fuzzy inference system (ANFIS) structure for prediction.

Figure 2 illustrates the architecture of ANFIS which consists of six layers with two input variables ($x_{1},x_{2}$) and one output (y). The six layers include one input layer, four hidden layers, and one output layer to show the fuzzy set theory.

Each layer performs a particular task to forward the signals. The layer features are presented as bellow.

The first layer shows the input layer of the ANFIS model and the neurons simply transmit the external input signals to the next layer.

A fuzzification neuron gives an input signal, and determines the degree to which this signal belongs to the neurons fuzzy set and this duty is related to the second layer of fuzzy rules.

In the third layer, the output signal is based on the first and second layers. Thus, the input layers in the third layer is the signal from fuzzification neuron “c”, which is obtained from the second layer and is also related to neuron “i” in the third layer.

The fourth layer, i.e., the third hidden layer, is the normalization layer. Each neuron in this layer receives signals from all rule neurons in the third layer and then normalize the data.

The fifth layer is the defuzzification layer. Each neuron in this layer is connected to the respective normalized neuron in the fourth layer and also receives initial input signals. The weighted consequent value is determined in this layer. In the sixth layer, there is only one neuron in this layer to calculate the sum of outputs of all defuzzification neurons in the fifth layer and consequently the overall ANFIS output “y” can be produced and expressed in Equation (3):

(3) $$y=\sum_{i=1}^{n}x_{i}$$

A multi-criteria assessment was conducted to evaluate the success of each model. The estimation capability of each model was evaluated using the Root Mean Square Error (R-MSE), and determination coefficient (R^2^) [36].

The determination of coefficient (R^2^) method is a popular method and is well-known for its capabilities in predicting and modelling materials. The R^2^ method is currently used to assess concrete properties, such as compressive strength, elastic modulus and water absorption. In this research, a total of eight factors including cement, metakaolin, nano-CuO, water, PP, sand, SP and age were considered as inputs to predict the compressive strength, flexural strength and water absorption of mortar as output layers using ANFIS method with MATLAB. The proposed model were developed and tested against the results derived from 52 strength tests (obtained from compressive and flexural strength tests at 28 and 90 days), and also 26 water absorption tests at 28 day.

**Figure 3:**
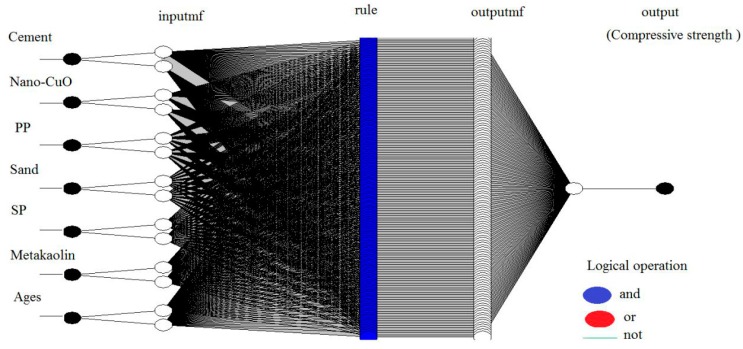
ANFIS structure for prediction.

After testing different functions in the FIS structure of MATLAB, the best values of output layer, the triangular-shaped membership function (TRIMF) and the linear membership function (MF) were selected with 2 number of MF for all of seven inputs using the hybrid train FIS with seven epochs to predict the compressive strength. For other outputs (such as water absorption and flexural strength), a triangular-shaped membership function (TRIMF) was used. However, the numbers of MF functions that obtained with trial and error processes were varied between 3 and 5 for each input variable in the model. Figure 3 indicates one of the FIS model structures which was built with MATLAB software. In this model the compressive strength was the output layer. In the present study, the ANFIS was trained, tested and validated for prediction of compressive strength, flexural strength and water absorption of cement mortars. The linear least square fit line, its equation (the relationship between predicted value and experimental value) and R^2^ are given in Table 5. For the training, testing and validation data, the best value of R^2^ was approximately 1 in the ANFIS model. The minimum values of R^2^ were 90% for checking set. All R^2^ values showed that the proposed ANFIS models are suitable and can be used to predict output layers such as compressive strength, flexural strength and water absorption of mortar.

#### 2.2.2. Pegasus (Primal Estimated Sub-Gradient Solver for SVM)

Support vector machines (SVMs) are considered the most effective and popular classification learning tool. The duty of learning a support vector machine is based on a constrained quadratic programming problem. This approach seems to be successful in solving the classification problem. This method is based on the minimization of the objective function, which involves empirical risk and keeping a low complexity of the classifier, simultaneously. In this study, the Pegasos used was based on an optimization problem in primal weight space and described as follows:

(4) $$\min\left(w\right)=\frac{\lambda }{2}{\left|w\right|}^{2}+\frac{1}{m}\sum_{\left(x,y\right)\in s}l\left(w;\left(x,y\right)\right)$$

Subject to the following equality constraints:

(5) $$y_{i}=w^{T}\varphi \left(x_{i}\right)+b\;i=1,2,\ldots .,n$$

As mentioned earlier, eight inputs (cement, metakaolin, nano CuO, water, PP, sand, SP and ages) were used to predict the mechanical properties of mortar (output layer). These input values were assumed to be x_i_ in Equation (5), then, by determination of the value of w, the value of b (output layer) would be estimated.

##### Using Mini-Batch Iterations to Implement Pegasos

In this study, it was assumed that a more general algorithm which utilized k ∈m as members of training data and A_t_ as subset data for each iteration (t). The subset was variable and chosen randomly after changing the iteration. The f (w) is an objective function in which w was minimized and determined in Equation (4) and f(w;At) was obtained for each k (members) and At (subset) with Equation (5) and expressed as follows:

(6) $$f\left(w;A_{t}\right)=\frac{\lambda }{2}\;{\left|\left|w\right|\right|}^{2}+\frac{1}{k}\sum_{i\in \mathrm{At}}l(w;(x_{i},y_{i})),\;l(w;\left(x_{i},y_{i}\right)=\max\left\{0,\;1-y\langle w,x\rangle \right\}$$

The suggested value of λ was used from previous research which is expressed as $\frac{1}{n}$ where n is the number of training data [37]. The goal of this method was to determine the optimum values of w and b for Equation (2) based on optimization of ∈ in Equation (7) which

(7) $$f\left(w_{t+1}-w_{t}\right)<\in$$

**Figure 4:**
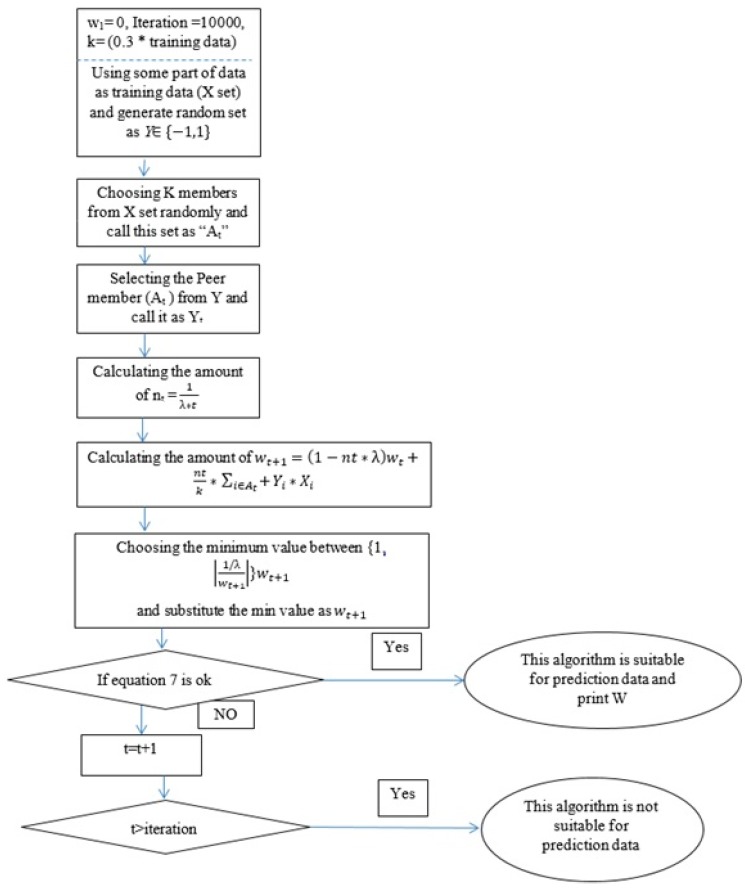
Pegasos step for solving prediction problem.

Figure 4 shows the steps of Pegasos method and the amount of w obtained after passing these steps. Eventually, some data were selected randomly for testing algorithm and evaluated the performance of algorithm with testing and training sets. The predicted values obtained from ANFIS and Pegasos models are illustrated in Figures associated with each test results.

## 3. Results and Discussion

### 3.1. Compressive Strength

**Figure 5:**
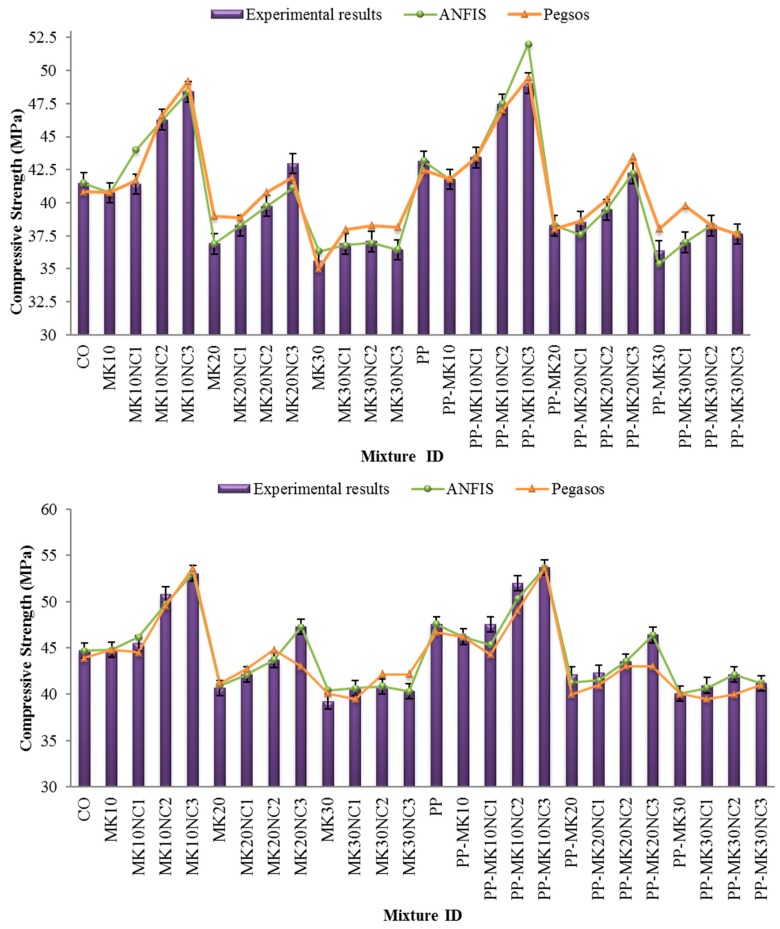
Compressive strength results at: 28 days (**top**); and 90 days (**below**).

The 28-day and 90-day compressive strength results of various mixes are shown in Figure 5.

**Figure 6:**
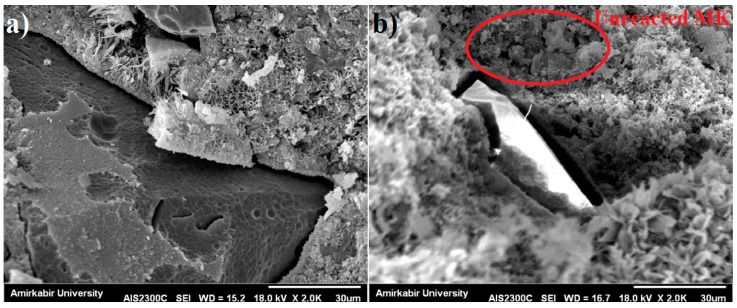
SEM images of: (**a**) CO sample; and (**b**) MK30 sample.

As can be seen in Figure 5, both 28- and 90-day compressive strength decreased as the amount of MK content increased. The strength of concrete containing MK depends on many factors, such as the quality of material’s composition, fineness, specific surface area, etc. [38]. The reduction in compressive strength could be due to two possible reasons. First, there was inadequate C–H to react with the MK at a high content rate, for example in the case of MK 20, some of the MK did not react and acted only as filler [3], as illustrated in the SEM image in Figure 6b. Second, the excess MK needs more water, leading to an insufficient development of hydration due to the inadequate amount of water. The higher level of the pozzolanic reaction in cement pastes with a lower replacement level might be attributed to a higher concentration of C–H available for the pozzolan to react with [39].

However, after 90 days of curing, the compressive strength of MK10 is slightly higher than that of control specimens. This increase is a result of the increased pozzolanic activity of MK in concrete during later ages. This can be explained by the chemical and pozzolanic properties of this material, which result in higher reactivity [40]. The calcium silicate hydrates (C–S–H) produced by pozzolanic activities, as well as the filling behaviour of MK at later ages, densify the hardened cement paste and block the capillary pores’ connection, so the pore structure of concrete is refined [2,40,41,42]. Furthermore, the Si and Al released from the MK are ascribed to the major aluminosilicate purities of this mineral, as seen in the chemical composition in Table 1. They could create suitable conditions for formation of certain amounts of C–A–S–H-like phases of binding gels, despite the low proportions of MK. In addition, the MK particles could foster the aluminosilicate ratio similar to the filler effect.

According to previous studies, significant reaction between MK and Portlandite (CH) was shown within the interfacial transition zone (ITZ) between aggregate and cement paste. It was reported that the ITZ with a high concentration of large and aligated CH crystals would lead to localized areas of boosted porosity and lower strength [43,44]. Figure 6 shows the SEM micrographs of cement mortar containing 30 wt % MK compared to the control sample. As shown in Figure 5b, the microstructure of cement matrix with MK was more porous containing a large amount of needle-like hydration products cemented together. Moreover, the gap at the ITZ between the aggregate and cement paste found in MK cement sample was much bigger when compared to the control sample, and consequently the cement mortar containing MK showed a reduction in strength.

**Figure 7:**
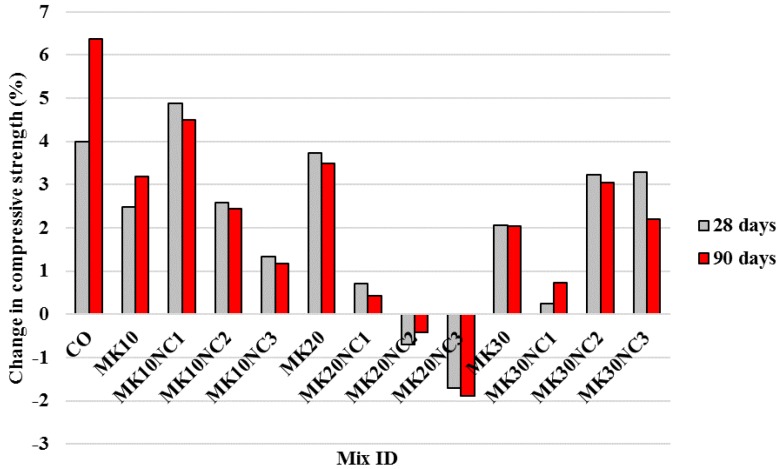
Percentage change in compressive strength of samples by the addition of fibres at 28 and 90 days with similar volumes of metakaolin and nano-CuO.

Figure 7 also shows that the compressive strength of cement mortars generally increased slightly when 0.3% PP was used. Although the strengths for mixtures containing 20% MK and 2% or 3% NC were found decreased in comparison with mixes without PP, the average strength for all samples only increased by 2% at both 28 and 90 days. The strength improvement is considered insignificant and the results are similar to the findings of previous studies with other pozzolans where a significant improvement in compressive strength has not been seen [45,46]. Compared to the CO sample, the incorporation of 3% NC further increased the strength of samples containing 10% MK up to 17% and 19% at 28 and 90 days, respectively. Samples containing 10% MK and PP also showed an increase up to 14% and 13% at 28 and 90 days, respectively. It is worth noting that samples containing 10% MK and 0.3% PP gave the highest compressive strength among all the specimens tested.

**Figure 8:**
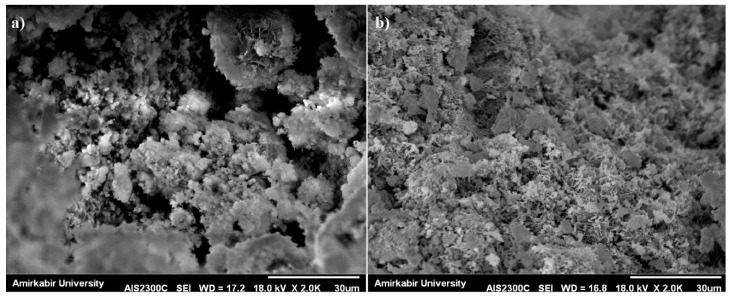
SEM images of: (**a**) MK10 sample; and (**b**) MK10NC3 sample.

Apparently, the inclusion of nano-CuO led to a significant strength improvement. The reasons can be explained by the following three-stage mechanisms:

- Filling property: NC can act as a filler to improve the density of mortar resulting in a significant reduction of porosity. Figure 8 shows the SEM micrographs of MK10 and MK10NC3 samples. As shown in Figure 8b, the microstructure of cement matrix containing NC was more compact and the porosity was significantly reduced. The SEM results confirmed that NC, having the filling ability property, can fill the porosity in cement paste and make a denser cement matrix.
- Acting as a nucleus: In the structure of the C-S-H gel, the nanoparticles can act like a nucleus forming an extremely strong bond with C-S-H gel particles [36]. Thus, when nanomaterials are uniformly dispersed in cement, they can promote the cement hydration due to their high reactivity, results in improvement of mechanical properties and durability of mortars.
- Crystal-formation control: If the amount of nanoparticles and their spacing are appropriate, the formation process of Ca(OH)_2_ crystals in the transition area can be reduced [36]. Therefore, with increase of NC up to 3%, the compressive strength raised except for the samples containing 30% MK. It can be stated that NC can lead to a denser structure with less porosity when an appropriate amount is added.

It can be concluded that addition of up to 3% NC to mortars is recommended. Similar suggestion was reported by Nazari et al. [20]. Nanoparticles improved the structure of the aggregates’ contact area, thus they can make a proper bonding between aggregate and paste, and result in a reduction of porosity in the interfacial transition zone (ITZ) between aggregate and paste [47].

It was observed that the addition of 3% NC and 10% MK enhanced the density and uniformity of specimens. It might be due to the accelerated cement hydration products which are associated with the high silica and alumina contents in MK and high free energy of NC. However, samples of MK30NC3 also indicated a satisfactory mechanical performance, particularly given that they are more environmental-friendly by consumption of more MK and using less cement content in concrete.

Figure 5 also presents the predicted results using the ANFIS and Pegasos models based on the statistical values. The values of R^2^, mean absolute percentage error (*MAPE*) and the relative root–mean–squared error (*RRMSE*) were reported for compressive strength at 28 days and 90 days are shown in Table 5. The results of ANFIS and Pegasos models showed that these two models are able to predict the strength values close to the experimental ones. Noteworthy, the prediction values for training set in ANFIS model were more accurate in comparison to the Pegasos model. On the contrary, the values obtained from Pegasos for testing data were closer to the laboratory test results compared to the ANFIS model.

### 3.2. Flexural Strength

**Figure 9:**
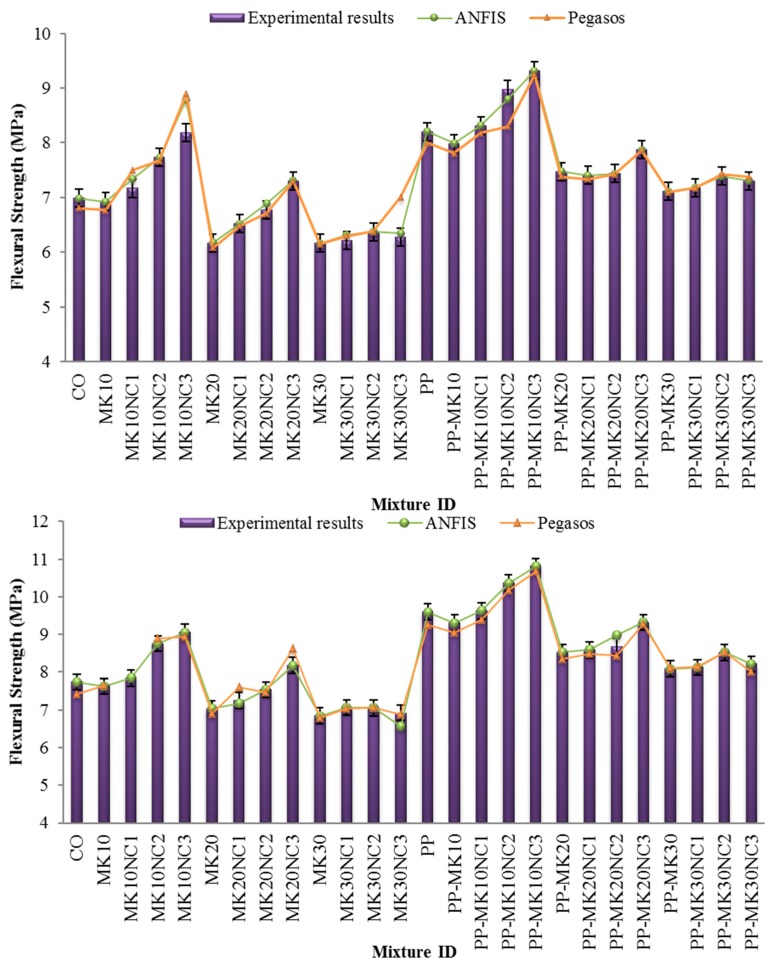
Flexural strength of mortars at: 28 days (**top**); and 90 days (**below**).

Figure 9 shows the flexural strength test results of samples incorporating NC, MK and PP.

Similarly, the flexural strength of mortar generally decreased with increasing MK content, however the strengths improved considerably with the increase of NC content. It is believed that CuO nanoparticles can provide interlocking effects between the slip planes, and thus improving the toughness and flexural strengths of cement-based materials [31]. Moreover, NC and MK together can improve the microstructural properties of cement paste due to their filler and pozzolanic effects, respectively. Besides, the enhancement of flexural strength may be due to the quick consumption of Ca(OH)_2_ with the high reactivity of nanoparticles during the hydration process particularly at early ages. Similar results were reported by Mohseni et al. [8] indicating that the flexural strength increased with increasing NC content up to 3%. However, any further increase in NC content showed insignificant strength improvement.

**Figure 10:**
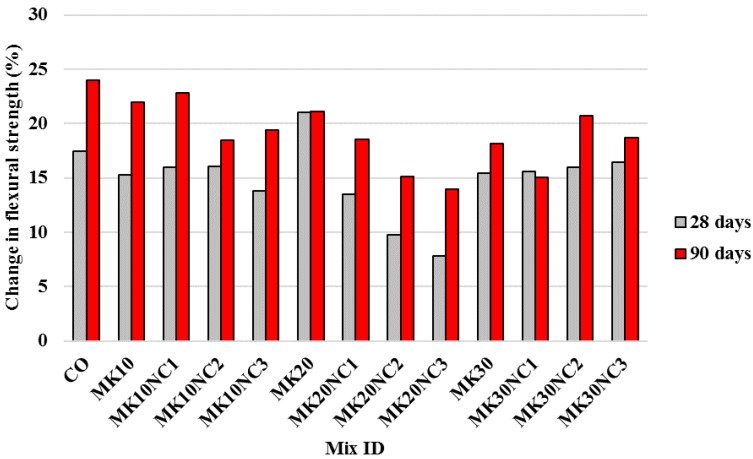
Percentage change in flexural strength of samples by the addition of fibres at 28 and 90 days with similar volumes of MK and NC.

**Figure 11:**
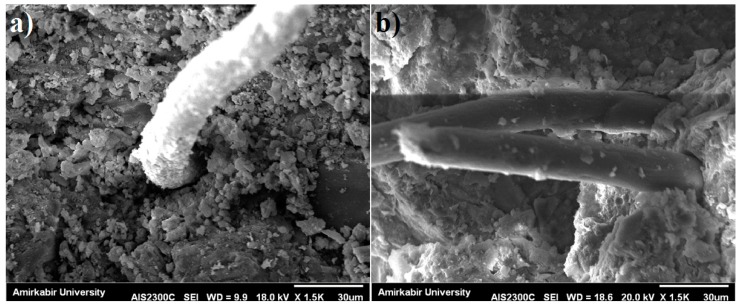
SEM image of: (**a**) PP; and (**b**) PP-MK10NC3.

Significant improvement can be seen when PP fibres were used as well (Figure 10). Compared to the samples without PP, the samples with PP showed an average increase of flexural strength by 12.7% and 17.4%, at 28 and 90 days, respectively. In addition, the flexural strength of mortar containing PP tended to increase with increasing NC content. This strength enhancement may be due to the improved microstructural interface between fibre and matrix, so the pull-out resistance of fibre was increased. Figure 11 shows the SEM micrographs of PP and PP-MK10NC3 samples. In PP-MK10NC3, there were more cement hydration products adhered around the fibres, accompanied with a more compact microstructure due to the filling ability of nanoparticles. This would improve the fibre–matrix interface, and thus enhance the load transfer between the cement matrix and fibres, leading an improvement in flexural strength of mortar.

Moreover, several micro cracks can be found at the cement matrix. Such cracks formation is mainly due to the plastic shrinkage in pre-hardening state as well as drying shrinkage in hardened concrete. As discussed in previous studies, by the addition of fibres, a strong bond between the fibre and cement paste will be formed, which would help restrain crack formation and propagation [48].

Figure 8 also shows the prediction results obtained from ANFIS and Pegasos models, the results obtained from these two models are very close to the laboratory test results.

### 3.3. Water Absorption

**Figure 12:**
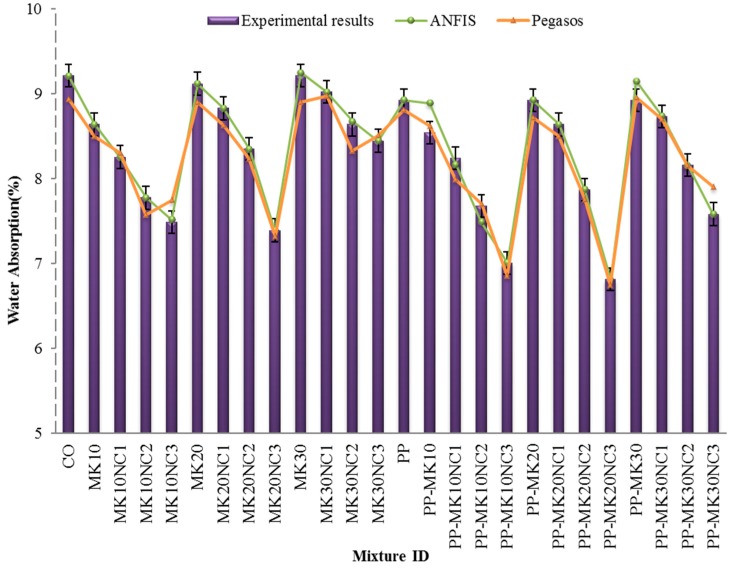
Water absorption of mortars at the age of 28 days.

The water absorption results for all specimens are presented in Figure 12. It can be seen from the figure that the water absorption of mortar samples decreased with the increase of MK content up to 10%. However, the addition of more MK (i.e., 20% and 30%) did not have remarkable impact on the water absorption. Previous research indicated that water absorption of concrete mixtures decreased with MK content up to 10% [1]. The main reason is due to the modification of pore size as a result of pozzolanic reaction.

Figure 12 also shows the water absorption decreased with increasing NC content. It is worth noting that the incorporation of NC in mortars can reduce the water absorption compared with the control sample (CO). This reduction is mainly due to the positive effect of nanoparticles in filling the micro-pores in the matrix as mentioned above. The interfacial transition zone in the mortar was improved due to the pozzolanic reaction as well as the filler effect of NC, and thus decreasing the water absorption.

**Figure 13:**
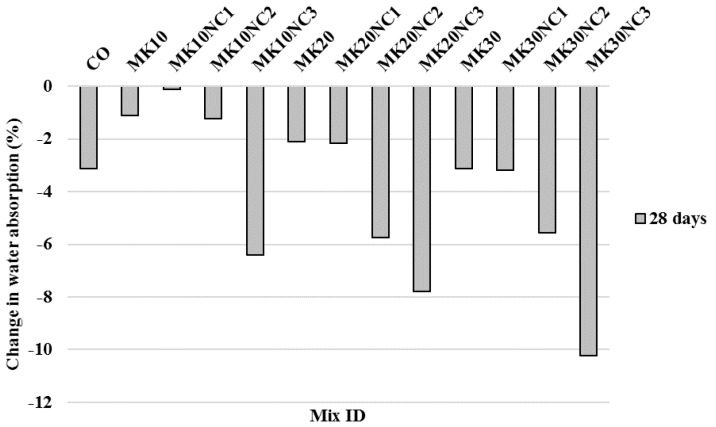
Percentage change in water absorption of samples by the addition of fibres at 28 days with similar volumes of MK and NC.

Furthermore, the addition of PP fibres apparently can reduce the water absorption as shown in Figure 13. For example, although no decrement of water absorption for mix PP-MK10NC1 was observed, a reduction of 10.23% for mix PP-MK30NC3 was observed compared to the specimens without fibre but with the same amounts of MK and NC.

The reduction in water absorption observed for the sample with 3% NC is attributed to the formation of jelly-like silicate hydrate, which fills the pores [38]. The reaction mechanism was derived from cement hydration with the presence of water. This reaction forms additional cementitious aluminium containing C-S-H gel, together with crystalline products [38]. The same conclusion on concrete without MK and NC can be drawn based on the results reported by Gong et al. [49]. The reduction of water absorption in samples including MK pozzolans and NC materials is generally associated with the decreased ratio of porosity (Figure 8), which reduces the pore water solution in specimens. The influential role of the pozzolanic materials such as MK on the water absorption properties of mixtures can be explained by the following factors. First, the pozzolanic materials act as a filler, hence reducing the average pore size and resulting in a less permeable paste. Moreover, the ITZ improvement occurs in samples because of the pozzolanic reaction as well as filler effect of MK and NC particles, respectively.

Figure 12 also shows the values of water absorption predicted by ANFIS and Pegasos models. Generally, the predicted results are in good alignment with the experimental results.

## 4. Accuracy of Predicted Methods

In this study, eight inputs (cement, metakaolin, nano CuO, water, PP, sand, SP and ages) were used to predict the properties of mortar and compared with the test results. The proposed model were developed and tested against a set of data derived from 52 concrete strength tests and 26 water absorption tests. The prediction accuracy of method was evaluated based on the mean absolute percentage error (*MAPE*), the relative root mean squared error (*RRMSE*) and the absolute fraction of variance (R^2^):

(8) $$MAPE=\frac{1}{n_{t}}\sum_{i=1}^{n_{t}}100\times \left|\frac{y_{i}-{\bar{y}}_{i}}{y_{i}}\right|$$

(9) $$RRMSE=\sqrt{\frac{n_{t}\sum_{i=1}^{n_{t}}{\left(y_{i}-{\bar{y}}_{i}\right)}^{2}}{(n_{t}-1)\;\sum_{i=1}^{n_{t}}y_{i}^{2}}}$$

(10) $$R^{2}=1-(\frac{\sum_{i=1}^{n_{t}}{\left(y_{i}-{\bar{y}}_{i}\right)}^{2}}{\sum_{i=1}^{n_{t}}{\bar{y}}_{i}^{2}})$$

where *y* and $\bar{y}$ are laboratory and predicted values, respectively; and *n_t_* is the number of samples. Smaller values of *RRMSE* and *MAPE*, and larger R^2^ indicate higher prediction accuracy.

Table 5 shows the statistical values of *MAPE*, *RRMSE* and R^2^ between the test and predicted results for compressive strength, flexural strength and water absorption.

Based on the results shown in Table 5, it can be stated that the ANFIS and Pegasos models were able to predict the properties of mortar very close to the experimental values. Compared to Pegasos model, ANFIS model gave the best performance in predicting compressive and flexural strengths. The corresponding values of R^2^, *RRMSE* and *MAPE* in the best performance were 1, 2.91 × 10^−6^ and 0.046, respectively, for compressive strength; 1, 0.0068 and 0.013, respectively, for flexural strengths; and 1, 1.18 × 10^−5^ and 0.004, respectively, for water absorption.

**Figure 14:**
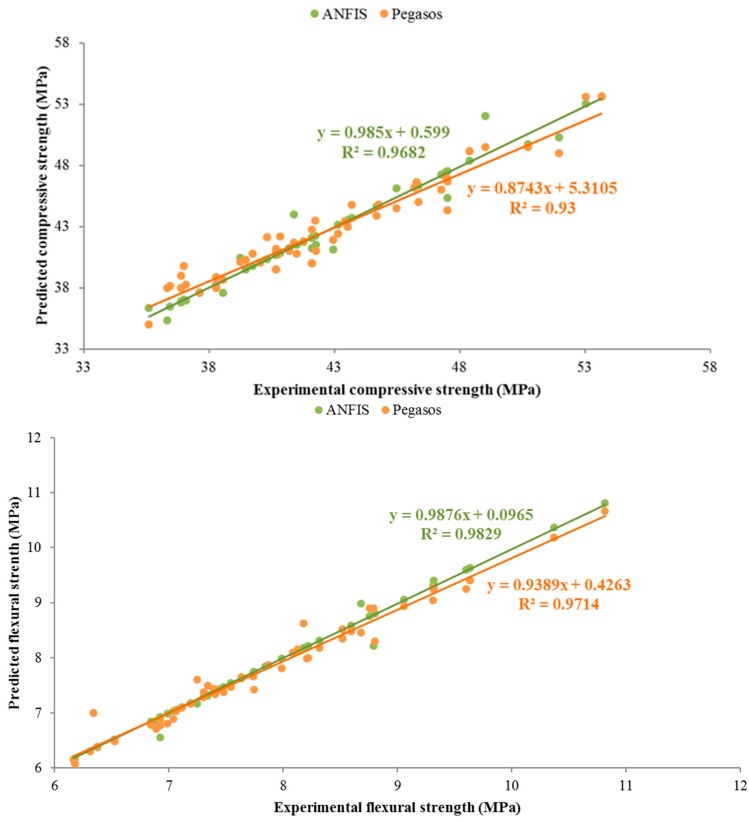

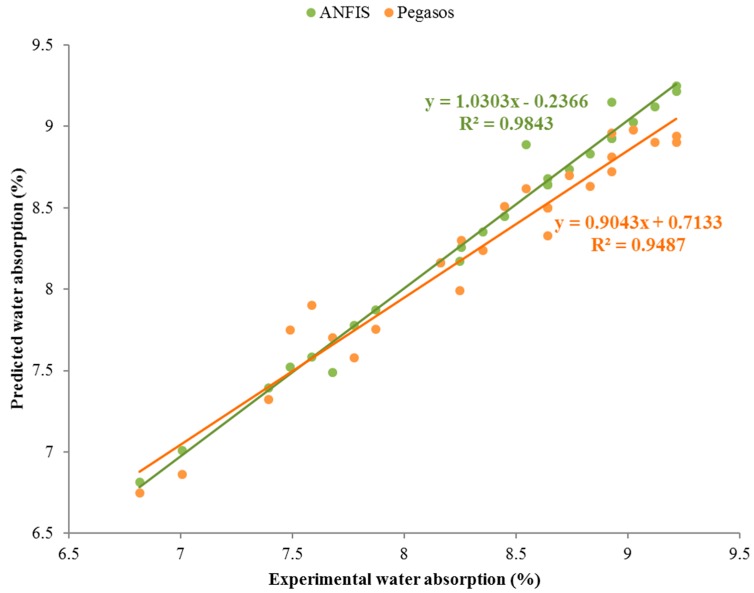
Correlation between experimental and predicted results.

Figure 14 illustrates the correlation between the experimental and predicted results by ANFIS and Pegasos. As is clearly shown, ANFIS and Pegasos methods are in close agreement with the experimental results. In addition, the values predicted by ANFIS are higher than those of Pegasos for all of the output layers (compressive strength, flexural strength and water absorption). The *R*^2^ values are more than 0.93 for both models, thus these two methods are practical and capable of accurately predicting the experimental results. Overlapping predicting values in ANFIS with Pegsos model indicates that predicted values during the training process in ANFIS model is so close to Pegasos model results. These two models have a little error to predict validating and testing set, that is why the gradient of trend ling in two model are different. Furthermore, the gradients of the two trend lines are almost one. Overlapping data in ANFIS and Pegsos models in flexural strength results are more than that for compressive strength. R^2^ value also increased in flexural strength results in comparison with the compressive strength. Therefore, the predicted results are more accurate.

Using either ANFIS or Pegasos methods has some benefits, which are mentioned as follows:

- Using ANFIS can get estimated results that are closer to the experimental results than Pegasos model.
- Using Pegasos model as an algorithm can obtain upper and lower bounds for each predicted data, while using ANFIS can only lead to one mean value, so user does not have any tolerance to report the data.
- Using ANFIS model requires obtaining the function, number of epoch and hidden layers with trial and error processes, while trial and error is not used in Pegasos algorithm.
- The speed of prediction process decreases with increasing number of data and input layers in ANFIS method and sometimes more time is required to run model when the hidden layers increase, however it is not an issue in Pegasos algorithm.

## 5. Conclusions

Based on the results obtained, the following conclusions are drawn:

- The compressive and flexural strengths decreased with increasing MK content at both 28 and 90 days.
- Using of 0.3% PP fibres improved the compressive strength slightly. The average compressive strength for all samples increased by 2% at 28 and 90 days which is negligible.
- Compared to the CO sample, the incorporation of 3% NC increased the strength of samples containing 10% MK up to 17% and 19% at 28 and 90 days, respectively.
- Significant improvement in flexural strength was seen when PP fibres were used. Compared to the samples without PP, the samples with PP indicated an average increase of flexural strength by 12.7% and 17.4%, at 28 and 90 days, respectively.
- Comparing with other samples tested, mortars containing 3% NC and 10% MK were considered as the most suitable mixtures for mechanical properties.
- It was observed that the water absorption of mortar samples decreased with the increase of MK content up to 10%. However, the addition of more MK (i.e., 20% and 30%) did not have remarkable impact on the water absorption.
- The addition of PP improved water absorption. The water absorption results showed that an addition of 0.3% PP fibres reduced the water absorption of mortar compared to the samples without PP.
- The water absorption results decreased with increasing the contents of nanoparticles and MK.
- SEM images illustrated that the morphology of cement matrix became more porous with increasing MK content, but the porosity reduced with the inclusion of NC. In addition, there were more cement hydration products adhered around the fibres, accompanied with a more compact microstructure due to the filling ability of nanoparticles. This could improve the fibre–matrix interface, and thus enhance the load transfer between the cement matrix and fibres, leading an improvement in flexural strength of mortar.
- Based on the statistical values of MAPE, RRMSE and R^2^, the ANFIS model showed the best prediction accuracy and can be used to predict the properties of fibre reinforced cement mortar accurately.

## Figures and Tables

**Table 1:** Chemical composition and physical properties of binder materials.

Chemical Analysis (%)	Cement	MK
SiO_2_	21.75	52.1
Al_2_O_3_	5.15	43.8
Fe_2_O_3_	3.23	2.6
CaO	63.75	0.2
MgO	1.15	0.21
SO_3_	1.95	0
K_2_O	0.56	0.32
Na_2_O	0.33	0.11
L.O.I	2.08	0.99
Surface area (BET) (m^2^/g)	0.31	2.54
Specific gravity	3.15	2.6

Note: The surface area was determined by gas adsorption method (BET).

**Table 2:** Properties of nano-CuO product.

Nanoparticles	Average Diameter (nm)	Specific Surface Area (m^2^/g)	Purity (%)
nano-CuO	20 ± 3	200	>99

**Table 3:** Properties of polypropylene fibre.

Unit weight (g/cm^3^)	0.9–0.91
Reaction with water	Hydrophobic
Tensile strength (MPa)	300–400
Elongation at break (%)	100–600
Melting point (°C)	175
Thermal conductivity (W/m/K)	0.12
Length (mm)	6
Diameter (μm)	20

**Table 4:** Mix details of mortars.

Sample ID	Cement (kg/m^3^)	MK (kg/m^3^)	NC (kg/m^3^)	PP (kg/m^3^)	Water (kg/m^3^)	Sand (kg/m^3^)	SP (kg/m^3^)
CO	450	0	0	0	220	1430	0.9
MK10	405	45	0	0	220	1415	1.9
MK10NC1	400.5	45	4.5	0	220	1410	1.9
MK10NC2	396	45	9	0	220	1400	1.9
MK10NC3	391.5	45	13.5	0	220	1395	1.9
MK20	360	90	0	0	220	1400	2.5
MK20NC1	355.5	90	4.5	0	220	1390	2.5
MK20NC2	351	90	9	0	220	1385	2.5
MK20NC3	346.5	90	13.5	0	220	1380	2.5
MK30	315	135	0	0	220	1380	3.5
MK30NC1	310.5	135	4.5	0	220	1375	3.5
MK30NC2	306	135	9	0	220	1370	3.5
MK30NC3	301.5	135	13.5	0	220	1360	3.5
PP	450	0	0	1.35	220	1430	1.75
PP-MK10	405	45	0	1.35	220	1415	2.75
PP-MK10NC1	400.5	45	4.5	1.35	220	1410	2.75
PP-MK10NC2	396	45	9	1.35	220	1400	2.75
PP-MK10NC3	391.5	45	13.5	1.35	220	1395	2.75
PP-MK20	360	90	0	1.35	220	1400	3.75
PP-MK20NC1	355.5	90	4.5	1.35	220	1390	3.75
PP-MK20NC2	351	90	9	1.35	220	1385	3.75
PP-MK20NC3	346.5	90	13.5	1.35	220	1380	3.75
PP-MK30	315	135	0	1.35	220	1380	4.25
PP-MK30NC1	310.5	135	4.5	1.35	220	1375	4.25
PP-MK30NC2	306	135	9	1.35	220	1370	4.25
PP-MK30NC3	301.5	135	13.5	1.35	220	1360	4.25

**Table 5:** Adaptive neuro-fuzzy inference system (ANFIS) and Primal Estimated sub-GrAdient Solver for SVM (Pegasos) results.

**ANFIS**	**R^2^ Values for: Training Set, Testing Set, and Validation Set**			
**Data Set**	**Training Set**		**Testing Set**	**Validation Set**
Compressive strength	R^2^	1	0.90	0.94
	RRMSE	2.96 × 10^−6^	9.48 × 10^−4^	4.8540 × 10^−4^
	MAPE	0.046	0.41	0.08
Flexural strength	R^2^	1	0.96	0.94
	RRMSE	6.98 × 10^−3^	0.016	0.0068
	MAPE	2.94	0.015	0.013
Water absorption	R^2^	1	0.93	0.97
	RRMSE	1.18 × 10^−5^	0.0023	0.0025
	MAPE	0.004	1.27	1.58
**ANFIS**	**The Relationship Between Predicted Values (y) and Experimental Data (x)**			
**Data Set**	**Training Set**		**Testing Set**	**Validation Set**
Compressive strength	y = 0.99x + 0.01		y = 0.99x + 0.23	y = 0.96x + 1.3
Flexural strength	y = 0.99x + 0.002		y = 1.04x − 0.37	y = 0.99x + 0.01
Water absorption	y = 0.99x + 0.02		y = 1.009x + 0.07	y = 1.17x − 1.43
**Pegasos**	**R^2^ Values for: Training Set, Testing Set, and Validation Set**			
**Data Set**	**Training Set**		**Testing Set**	
Compressive strength	R^2^	0.96	0.9	
	RRMSE	2.91 × 10^−4^	0.00052	
	MAPE	1.85	2.47	
flexural strength	R^2^	0.99	0.91	
	RRMSE	0.0017	0.005	
	MAPE	1.07	3.31	
Water absorption	R^2^	0.96	0.9	
	RRMSE	2.96 × 10^−6^	2.85 × 10^−3^	
	MAPE	0.00305	1.66	
**Pegasos**	**The Relationship between Predicted Values (y) and Experimental Data (x)**			
**Data Set**	**Training Set**		**Testing Set**	
Compressive strength	y = 0.91x + 4.23		y = 0.78x + 10.41	
Flexural strength	y = 0.97x + 0.12		y = 0.88x + 1.03	
Water absorption	y = 0.8x + 1.58		y = 0.92x + 0.47	
