# Correction: Ghanei et al. Effect of Nano-CuO on Engineering and Microstructure Properties of Fibre-Reinforced Mortars Incorporating Metakaolin: Experimental and Numerical Studies. *Materials* 2017, *10*, 1215

**DOI:** 10.3390/ma14113012

**Published:** 2021-06-02

**Authors:** Amir Ghanei, Faezeh Jafari, Mojdeh Mehrinejad Khotbehsara, Ehsan Mohseni, Waiching Tang, Hongzhi Cui

**Affiliations:** 1 Department of Civil Engineering, Mahmoudabad Branch, Islamic Azad University, Mahmoudabad 46456-55111, Iran; Ghaneiamir91@gmail.com; 2 Department of Civil Engineering, Malayer University, Malayer 65719-95863, Iran; faeze_jafari666@yahoo.com; 3 Centre for Future Materials (CFM), School of Civil Engineering and Surveying, University of Southern Queensland, Toowoomba, QLD 4350, Australia; Mojdeh.Mehrinejad@usq.edu.au; 4 School of Architecture and Built Environment, The University of Newcastle, Callaghan, NSW 2308, Australia; Ehsan.Mohseni@uon.edu.au; 5 Shenzhen Durability Center for Civil Engineering, Shenzhen University, Shenzhen 518060, China; h.z.cui@szu.edu.cn

The authors regret that Figures 6 and 11b in [1] were printed incorrectly. Therefore, the authors wish to make the following corrections to Figure 6 and Figure 11b. Since the analysis was conducted based on the following images, the correction does not affect the results and conclusions.

## Figures and Tables

**Figure 6:**
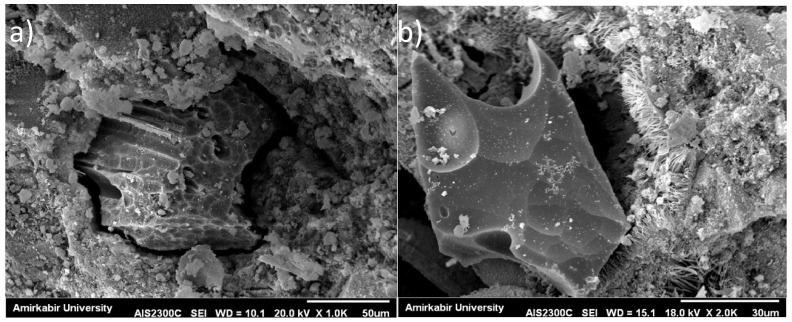
SEM images of: (**a**) CO sample; and (**b**) MK30 sample.

**Figure 11:**
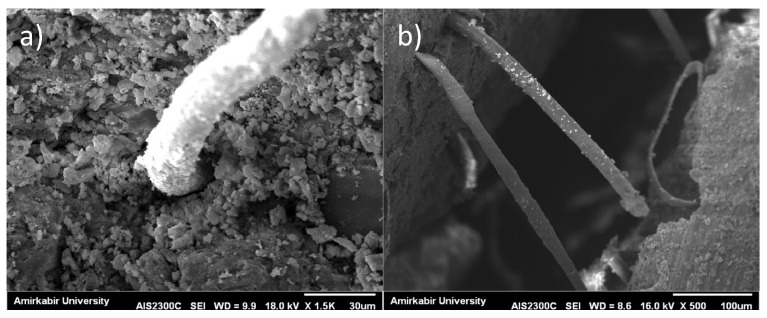
SEM images of: (**a**) PP; and (**b**) PP-MK10NC3.
